# Object Detection and Depth Estimation Approach Based on Deep Convolutional Neural Networks †

**DOI:** 10.3390/s21144755

**Published:** 2021-07-12

**Authors:** Huai-Mu Wang, Huei-Yung Lin, Chin-Chen Chang

**Affiliations:** 1 Department of Electrical Engineering, National Chung Cheng University, Chiayi 621, Taiwan; huaimu@godel.ee.ccu.edu.tw (H.-M.W.); lin@ee.ccu.edu.tw (H.-Y.L.); 2 Advanced Institute of Manufacturing with High-Tech Innovations, National Chung Cheng University, Chiayi 621, Taiwan; 3 Department of Computer Science and Information Engineering, National United University, Miaoli 360, Taiwan

**Keywords:** object detection, depth estimation, stereo vision, deep learning

## Abstract

In this paper, we present a real-time object detection and depth estimation approach based on deep convolutional neural networks (CNNs). We improve object detection through the incorporation of transfer connection blocks (TCBs), in particular, to detect small objects in real time. For depth estimation, we introduce binocular vision to the monocular-based disparity estimation network, and the epipolar constraint is used to improve prediction accuracy. Finally, we integrate the two-dimensional (2D) location of the detected object with the depth information to achieve real-time detection and depth estimation. The results demonstrate that the proposed approach achieves better results compared to conventional methods.

## 1. Introduction

Autonomous driving techniques [1,2,3] have been studied intensively for several decades. Because of the advances in sensor technology and the demands of commuters, manufacturers have expended considerable resources on developing autonomous vehicles. The Society of Automotive Engineers classifies five levels of automated driving, the third level of which is conditional automation, or self-driving under ideal conditions with limitations. This level has drawn much attention as developers attempt to implement effective detection and recognition of the surrounding environment (e.g., the road, traffic signs, other vehicles, and pedestrians) so that the vehicle can detect and recognize objects ahead and estimate their depth from a visual sensor.

Conventional approaches to object detection use multiple windows of varying sizes to slide repeatedly over images at fixed distances to detect objects of interest. Felzenszwalb et al. [4] presented a pedestrian detection approach that used a deformable part model with a histogram of oriented gradients and a support vector machine. Recently, the advent of convolutional neural networks (CNNs) [5,6,7,8,9,10,11] rapidly superseded traditional object detection. These deep neural networks hypothesize bounding boxes, extract features from them, and use high-quality object classifiers.

In this paper, we propose a real-time object detection and depth estimation approach using learning-based techniques for images acquired from a vehicle’s onboard camera. First, we present an improved object detection approach—in particular for small objects—and then use deep neural networks and epipolar geometry to create stereo images and generate depth maps. Our approach modifies the monocular depth estimation network [12] for binocular images and introduces a new correlation function to generate disparity maps through model training with some image reconstruction loss. Finally, the two-dimensional (2D) location is integrated with depth information to achieve effective object detection and depth estimation. This paper is organized as follows: Section 2 reviews related works. Section 3 is the proposed approach. Section 4 describes the implementation and results. Section 5 presents the conclusions.

## 2. Related Works

CNN-based object detection approaches [7,8,9,10,11] were primarily divided into two categories, namely, one-stage and two-stage detection. In two-stage detection, features were extracted from candidate regions and targets were classified. Such methods included the faster region-based CNN (Faster R-CNN) [7], region-based fully convolutional network (R-FCN) [8], and feature pyramid network (FPN) [9]. A network structure with heuristic sampling was used to target the class imbalance problem, and cascading can regress the parameters of the bounding box. Generally, CNN representation played a key role in these methods. The learned features were designed to encode highly discriminative and robust object characteristics with a moderate position bias. Several approaches were proposed to address these problems. For instance, ResNet and Inception both extracted features from deeper CNN backbones [13,14]. The FPN introduced a top-down architecture to construct feature pyramids and integrated low- and high-level information [9]. However, extracting such features from deeper neural networks led to high computational costs and networks with a low inference rate.

In a one-stage detection method, designed to be efficient and computationally low cost, candidate region extraction and target classification are performed in an end-to-end network, such as a Single Shot MultiBox Detector (SSD) [10] or a You Only Look Once (YOLO) [11]. To accelerate the detection phase, a single-stage framework was proposed and an object proposal generation was removed. YOLO [11] and SSD [10] have demonstrated the possibility of real-time processing with a clear drop of 10 to 40% of current two-stage solutions. RetinaNet [15] substantially improved the precision scores so that they become comparable to the highest scores reported for two-stage detectors. Unfortunately, these performance gains were credited to the deep ResNet-101 model [13], which greatly limited efficiency.

The SSD used multiple bounding boxes of different sizes to detect dense objects quickly and accurately. However, its small-vehicle detection performance was low because it ignored the smaller features between layers. The average precision (AP) and average recall for small objects in the Microsoft Common Objects in Context (COCO) dataset [16] were only 5.3 and 9.6%, respectively [10]. Other approaches for detecting small objects must be developed for particular applications. Because shallow convolutional networks produce feature and texture loss, previous approaches could not detect small objects effectively. To address this problem, RefineDet [17] was adopted as the main detection framework. It used the advantages of the Faster R-CNN and SSD and incorporated the FPN for shared features. It exhibited an AP of 25.6% on the COCO dataset over the original SSD and a high frame rate. In our approach, we used the global information in the convolutional layers to improve the transfer connection blocks (TCBs). The detection benchmarks were then evaluated based on the PASCAL Visual Object Classes (VOC) and COCO datasets [16,18].

For traditional stereopsis, environmental parameters were limited and certain parts of the left and right images were inconsistent because the angles at which the user viewed the images prevented the calculation of disparity values. To solve the two problems, several depth estimation approaches were proposed. The conventional stereo vision approaches [19,20,21,22] included CNN-based methods and video-based processing. Deep learning techniques markedly improved depth estimation performance on the KITTI dataset [23]. For different input sources, the existing networks were modified for single-view [12,24] and stereo-view depth estimation [19,25,26] with a multi-scale CNN and probabilistic graphical models.

Several approaches based on fully convolutional networks (FCNs) for view synthesis and depth estimation have been proposed, wherein ground-truth depth maps were not necessary in the training stage. In Deep3D [27], left images were input to binocular vision images to create the corresponding right images. The core idea was to use a single image to reconstruct stereopsis, with the disparity values of the single image predicted on the basis of the probabilities of such values occurring for each image pixel. Next, the disparity images were used to synthesize the right images. To predict more accurate stereo images in unsupervised monocular image depth estimation networks (e.g., Monodepth [12]), the consistency and gray-scale smoothness of the left–right stereo images and right–left stereo images were carefully considered before the loss function was modified. Unsupervised training-based depth estimation networks were subsequently introduced to solve these problems.

## 3. Proposed Approach

**Figure 1:**
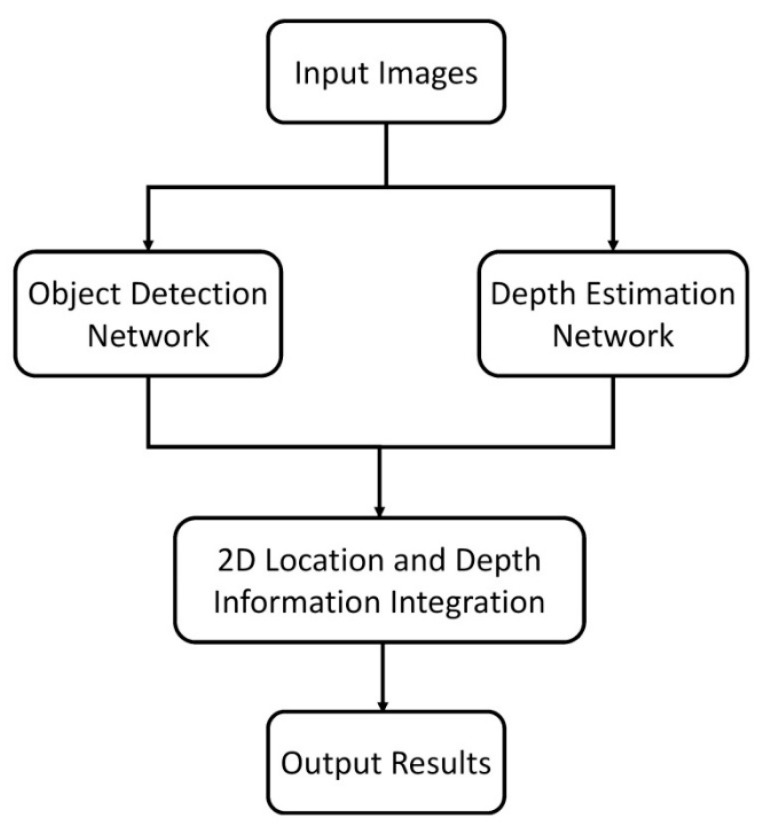
Flowchart of the proposed approach.

In the proposed approach, we presented a real-time object detection and depth estimation approach based on a light-network structure. Our approach consisted of two parallel modules: object detection and depth estimation. In the flowchart in Figure 1, input images were acquired from a vehicle’s onboard camera from which we presented an improved object detection approach. Then, we use deep neural networks and epipolar geometry to create stereo images and generate depth maps. We modified the stereo image network for disparity prediction and used the epipolar constraint to derive depth images from these disparities. Finally, we integrated the 2D location with the depth information and output the results for applications.

### 3.1. Object Detection

Object detection networks are plagued by problems such as high computational cost and inaccurate identification of small and faraway objects. Because small objects occupy a small space in the images, their detailed features are filtered out in the first few convolutional layers, and they are consequently ignored. To address this problem, we consulted the FPN, in which the feature information of all feature layers is shared and detailed patterns or context features are retained. Additionally, to reduce computational cost, we employ VGG16 as the backbone network.

RefineDet [17], which focuses on small-object detection and real-time computing capabilities, contains a network model that combines a two-stage and a one-stage detection network. It consists of two inter-connected modules: the anchor refinement module (ARM) and the object detection module (ODM). The ARM is used to remove negative anchors to reduce search space and roughly adjust the locations and sizes of anchors for better initialization. The ODM is used to regress correct locations of objects and predict multi-class labels based on the refined anchors. The TCBs are designed to fuse the information of the upper and lower convolutional layers between the ARM and the ODM. In our approach, we modified RefineDet [17] to improve small-object detection based on the following two improvements.

(1) Enhanced fine-feature extraction:

Inspired by single-shot face detection with feature fusion and segmentation supervision [28], we replaced element-wise addition with element-wise multiplication to prevent overflow. To suppress noise, we use the parametric rectified linear unit (PReLU) [29] as the activation function. The differences between correct classification and misclassification were strengthened and the lower computational cost improved object detection. This solved the problem of poor object detection for small and distant objects and high extraction rates in incorrect candidate regions.

(2) Shared global information with features of each pixel:

We imported global features, all feature maps could share the global information of other feature maps. Hence, we strengthened crucial features and suppress noise.

**Figure 2:**
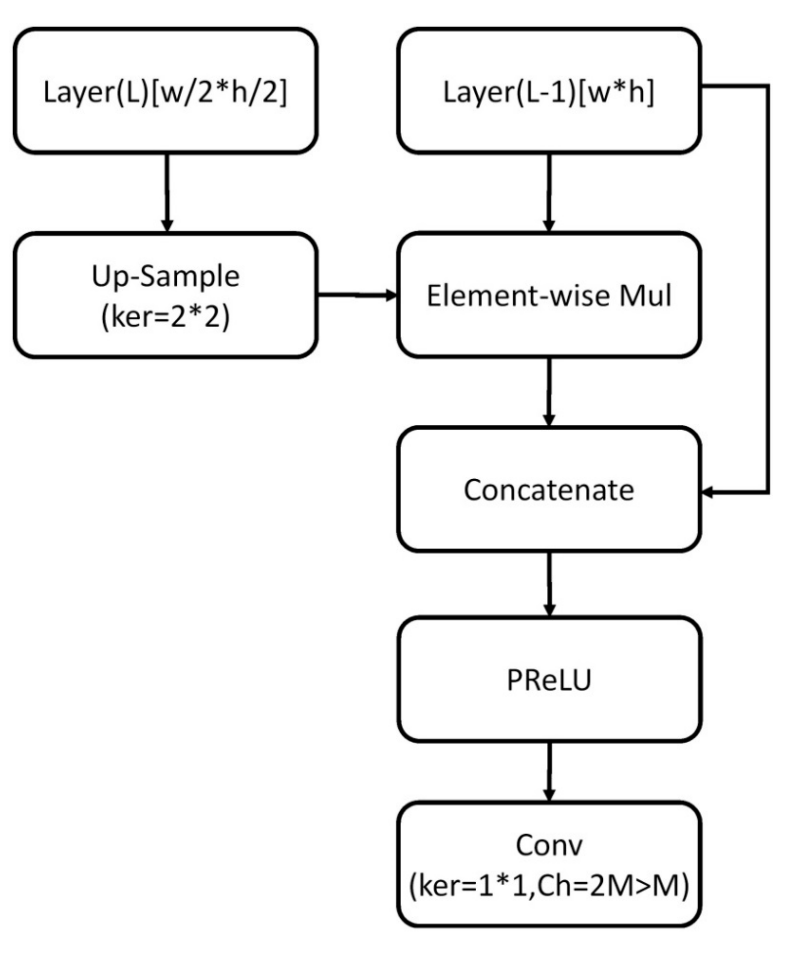
Flowchart of the improved TCB used for the modified RefineDet.

In the following, we introduced improvements for object detection in more detail. The proposed approach combined the concept of a two-stage detector into a one-stage network. We first extracted the object–agnostic region from the ARM and then used the ODM to classify the multi-scale object within the selected region. Finally, we added the TCBs to connect the feature maps to share information between low- and high-level layers. Figure 2 shows a flowchart of the improved TCB used for the modified RefineDet. We first up-sampled Layer (*L*) to match the dimensions between Layer (*L*) and Layer (*L*-1). Then, we replaced element-wise addition with element-wise multiplication. Third, transferred features were obtained by multiplying up-sampled Layer (*L*) and Layer (*L*-1) in the element-wise way. After that, we concatenated Layer (*L*-1) to the obtained transferred features. Finally, we applied the PReLU activation function before the convolutional layer.

**Figure 3:**
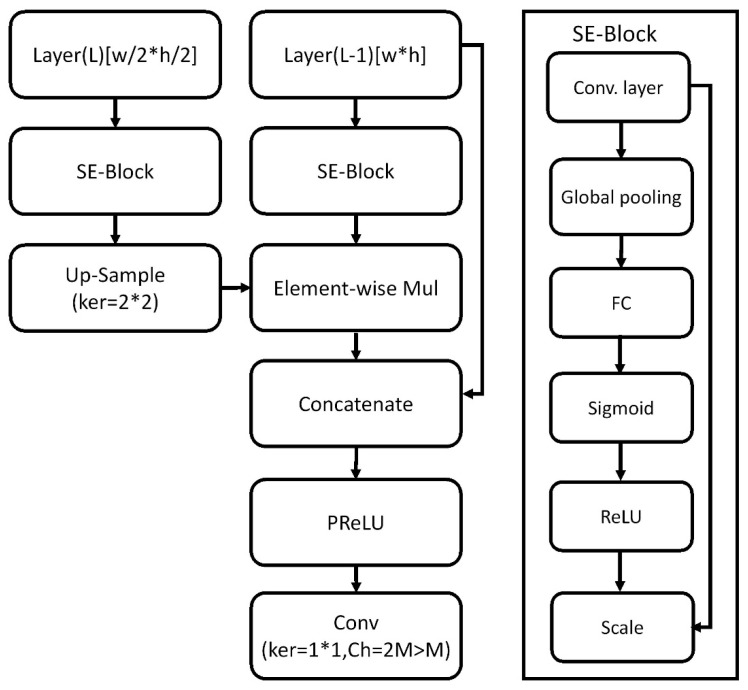
TCB block model with incorporated squeeze-and-excitation flow.

Moreover, we used the SENet [30] to enable the received global information in the feature maps to be shared with each cell. All feature maps shared the global information of other feature maps, strengthening crucial context features and suppressing noise. Figure 3 illustrates the TCB model with incorporated squeeze-and-excitation flow. For a convolutional layer, SE-Block proposes to share the global features by using global pooling. Then, SE-Block uses a fully connected (FC) layer, the Sigmoid function, and the ReLU function to limit model complexity. The final output of the SE-Block is obtained by scaling.

### 3.2. Depth Estimation

**Figure 4:**
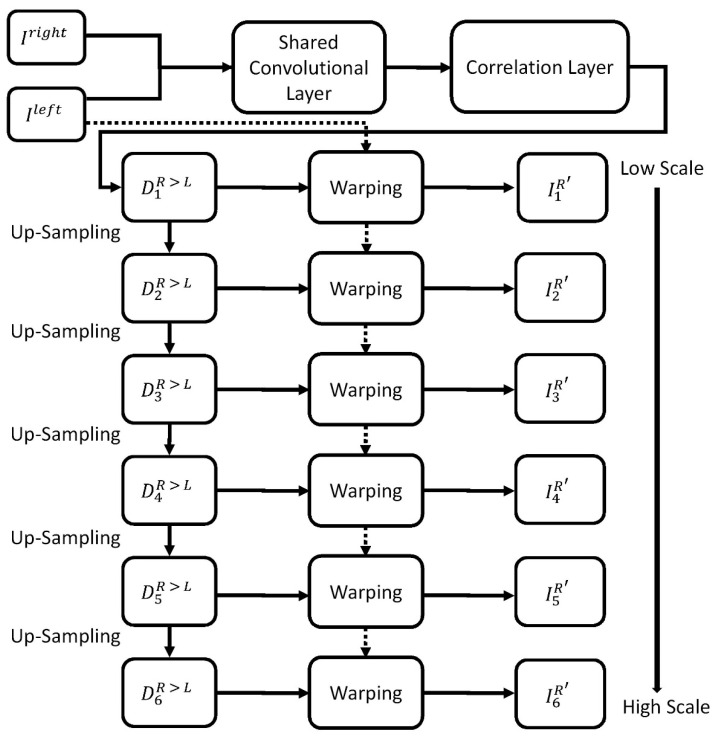
Modified network structure with binocular images. Stereo image pairs are used to generate the disparity maps.

For depth estimation, the proposed approach was based on Monodepth [12] and used stereo vision to predict the disparity maps [31,32]. The original Monodepth was designed for disparity estimation from monocular images but not binocular images. Hence, we modified the network structure for binocular images, as depicted in Figure 4. The improvements made to the depth estimation network architecture are as follows:

(1) Input layer: We input left and right images;
(2) Shared convolutional layer: With shared weights, we used the same convolution kernel to extract features of left and right images;
(3) Correlation layer: We use mathematical inner product operations to match the common regions between left and right feature maps;
(4) Disparity map prediction: We predicted all the possible disparity values for all matching points using a normal distribution method for six different scales; and
(5) Grayscale image reconstruction: We reconstructed the left and right images on the basis of the predicted disparity maps and the internal camera parameters for the six scales.

Moreover, we proposed a learning method to perform single-image depth estimation with a deep neural network despite the lack of ground-truth depth information. Compared with other approaches, the accuracy of the output disparities was lower for the single-image input. We therefore introduced a training loss to improve the robustness and consistency of the generated left and right images. We defined an image reconstruction loss function $L_{total}$ with the epipolar constraint for the disparity map generation as follows:

(1) $$L_{total}=\alpha_{ap}\left(L_{ap}^{l}+L_{ap}^{r}\right)+\alpha_{ds}\left(L_{ds}^{l}+L_{ds}^{r}\right)+\alpha_{lr}\left(L_{lr}^{l}+L_{lr}^{r}\right),$$

(2) $$L_{ap}^{l}=\frac{1}{N}\;\sum_{i,j}\alpha \frac{1-SSIM\left(I_{i,j}^{l},\;{\hat{I}}_{i,j}^{l}\right)}{2}+\left(1-\alpha \right)∥\left(I_{i,j}^{l},\;{\hat{I}}_{i,j}^{l}\right)∥,$$

(3) $$L_{ds}^{l}=\frac{1}{N}\;\sum_{i,j}|\delta_{x}d_{i,j}^{l}\left|e^{-∥\delta_{x}I_{i,j}^{l}∥}+\frac{1}{N}\;\sum_{i,j}|\delta_{y}d_{i,j}^{l}\right|e^{-∥\delta_{y}I_{i,j}^{l}∥},$$

(4) $$L_{lr}^{l}=\frac{1}{N}\;\sum_{i,j}|d_{i,j}^{l}-d_{i,j+d_{i,j}^{l}}^{r}|.$$

In Equation (1), $L_{total}\;$ consists of $L_{ap}^{l}$, $L_{ap}^{r}$, $L_{ds}^{l}$, $L_{ds}^{r}$, and $L_{lr}^{l},$$L_{lr}^{r}$ with weights $\alpha_{ap}$, $\alpha_{ds}$, and $\alpha_{lr}$. In (2), $L_{ap}^{l}$ indicates the structural similarity (SSIM) index consistency between the two left images (the original ground-truth image and the generated image); *N* is the number of pixels; and $I_{i,j}^{l}$, ${\hat{I}}_{i,j}^{l}$, and SSIM (·) represent the real image, generated image, and SSIM, respectively. In (3), $L_{ds}^{l}$ indicates the smoothness of the generated image for the surrounding pixels, where *δ_x_*, *δ_y_*, *d_i_*_,*j*_, and *I_i_*_,*j*_ represent the Gaussian standard deviation on the *x*-axis direction, the Gaussian standard deviation on the *y*-axis direction, the depth of the pixel, and the original image, respectively. In (4), $L_{lr}^{l}$ indicates the consistency between the two predicted disparity maps, where $d_{i,j}^{l}$ and $d_{i,j+d_{i,j}^{l}}^{r}$ are the left and right disparity values, respectively. Also, $L_{ap}^{r}$, $L_{ds}^{r}$, and $L_{lr}^{r}$ can be defined similarly;

Another component of our approach was the input of the image pair to the low-level layers of the convolutional network to determine common features between the left and right images. The features are then sent to DispNetC [19] for correlation prediction. In the network training stage, DispNetC extracts the inner product of the conjugate epipolar lines for feature matching. The correlation of two feature maps centered at $x_{l}$ in the left feature map and $x_{r}$ in the right feature map is defined by

(5) $$c\left(x_{l},x_{r}\right)=\sum_{o\in \left[-k,k\right]\times \left[-k,k\right]}<f_{l}\left(x_{l}+o\right),f_{r}\left(x_{r}+o\right)>,$$

where $f_{l}$ and $f_{r}$ are the left and right feature maps, respectively; *k* is a constant.

All possible disparities *D_i_*_,*j*_ in the image are predicted by the normal distribution

(6) $$\sum_{d}D_{i,j}^{d}=1,\;0<d<1,$$

where *d* is the probability of the matching point corresponding to each disparity value and

(7) $$D=\frac{f\cdot B}{Z},\;$$

where *f*, *B*, and *Z* are the focal length, stereo baseline, and depth, respectively. The disparity map is then converted to a gray-level image by

(8) $${\hat{I}}_{i,j}^{r}=\sum_{d}I_{i,j}^{d}D_{i,j}^{d},$$

and

(9) $$I_{i,j}^{r}=I_{i,j+D}^{l}.$$

Finally, the $L_{1}$ norm is used and the loss function for network prediction is calculated by

(10) $$L_{1}=∥{\hat{I}}^{r}-I^{r}∥.$$

## 4. Implementation and Results

This section reported the implementation and results of the proposed approach for object detection and depth estimation.

### 4.1. Implementation

Unlike the conventional approaches, this proposed approach did not require the parameters to be adjusted for different environments. Considering the computational constraint on mobile systems for vehicular applications, we used the same hardware platform (NVIDIA GTX 1080) to evaluate the performance of different algorithms. The stereo camera system used for data acquisition cost much less than light detection and ranging (LiDAR) or other time of flight (ToF) sensors. We performed quantitative analysis on several datasets and compared the proposed approach with previous methods. Datasets used for evaluation included PASCAL VOC [18], KITTI [23], BDD100K [33] and our own database. The software environment contained Ubuntu-16.04, Python 3.5, a Machine Learning API PyTorch graphics processing unit 0.40 [34], and a Tensorflow graphics processing unit 1.40 v. It was not easy to determine the parameters appropriately. In the experiments, these were set heuristically for the best performance. The training parameters were as follows: the learning rate, iteration, and batch size for the detection network were 0.01, 120,000, and 16, respectively; those for the depth estimation network were 0.01, 50, and 8, respectively.

### 4.2. Evaluation on Object Detection

**Figure 5:**
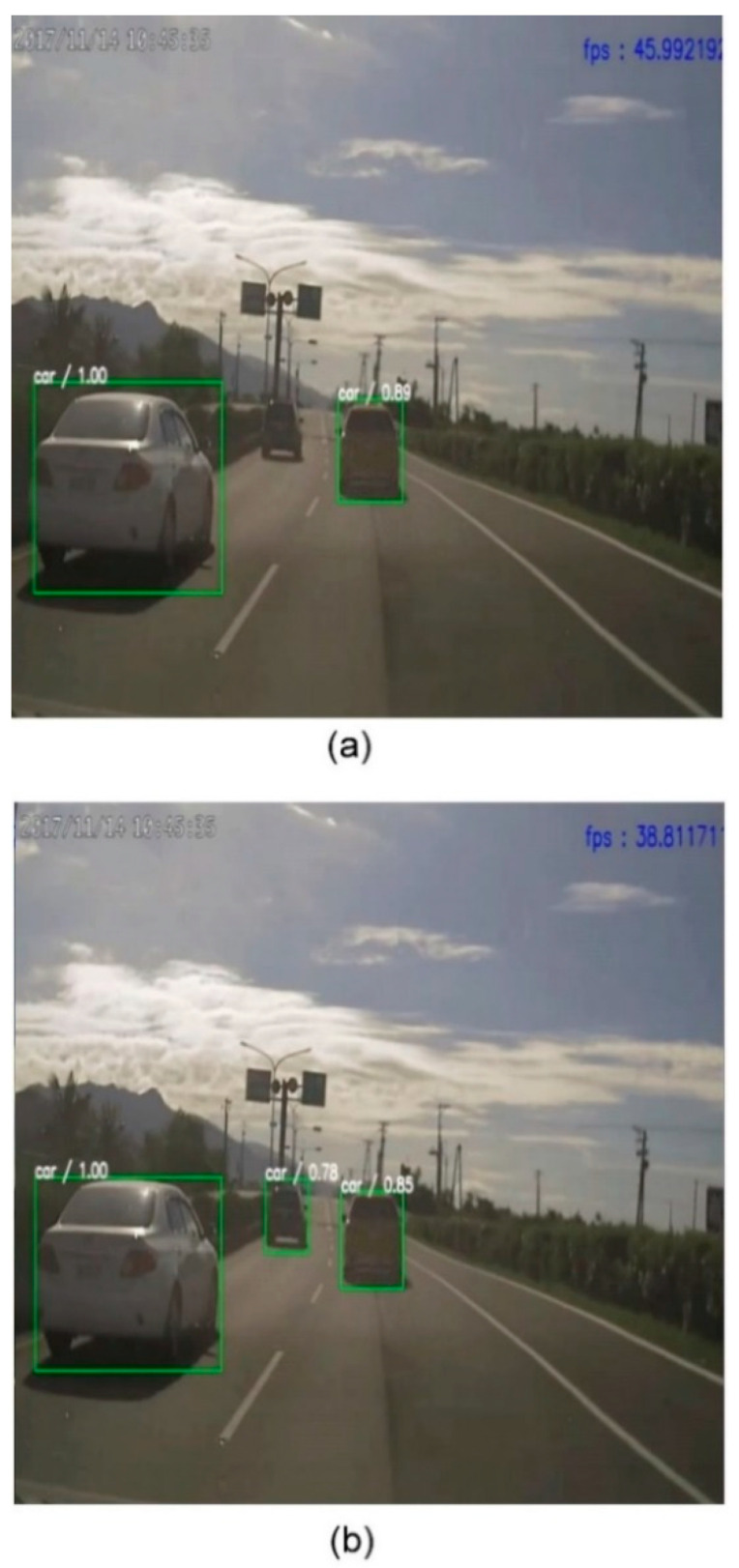
Comparison of the object detection results. (**a**) The resulting image is obtained from the original RefineDet. (**b**) The resulting image is the output of our approach.

For the detection network, the improved TCB structure over RefineDet increased the accuracy but not the network complexity as the processing frame rate was maintained at a stable value. Small object detection was also improved for faraway vehicles. As shown in Figure 5, the detection range was increased approximately from 25 to 50 m and the frame rates of the two methods were maintained in real time.

**Figure 6:**
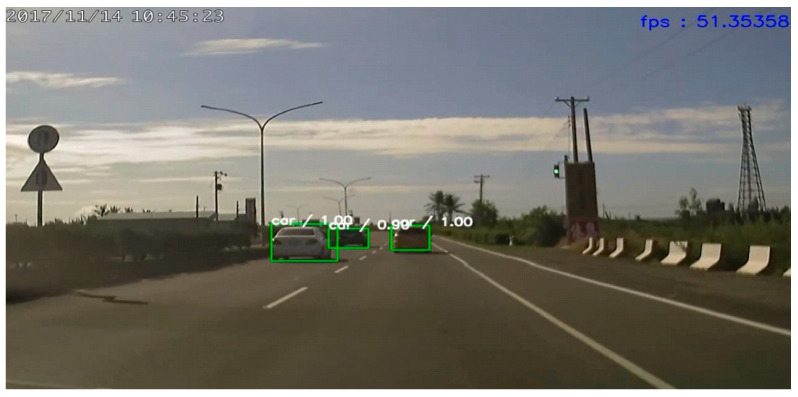
Reducing the number of classes to speed up object detection.

**Figure 7:**
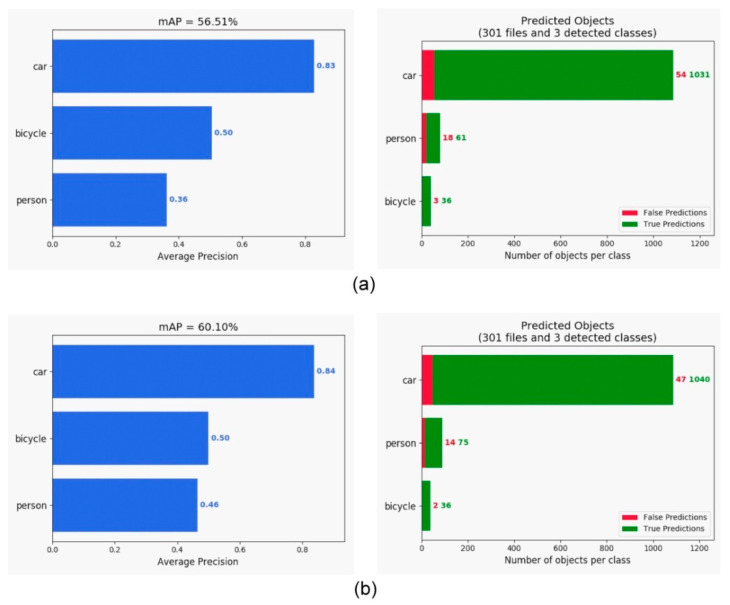
mAP comparison of the original RefineDet and our approach tested on the KITTI dataset. (**a**) Evaluation of RefineDet. (**b**) Evaluation of our approach.

**Figure 8:**
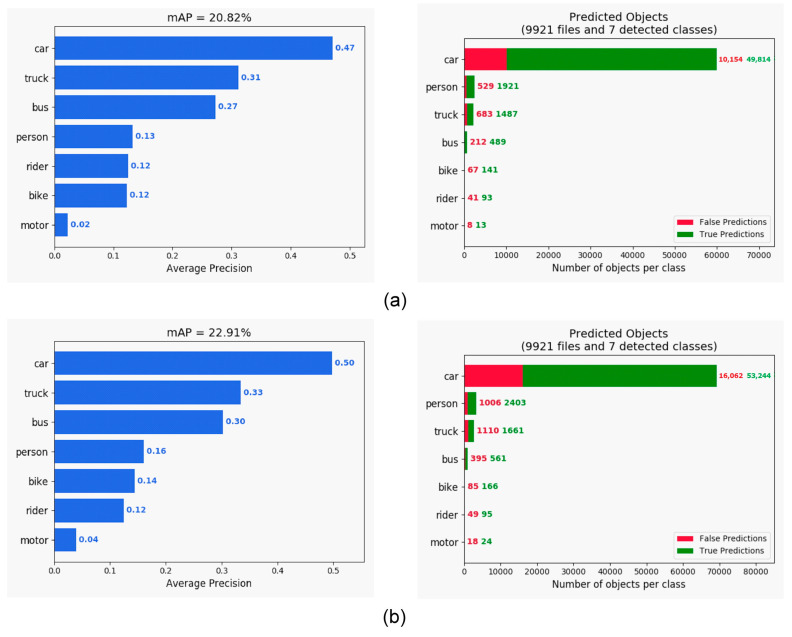
mAP comparison of the original RefineDet and our approach tested on the BDD100K dataset. (**a**) Evaluation of RefineDet. (**b**) Evaluation of our approach.

**Figure 9:**
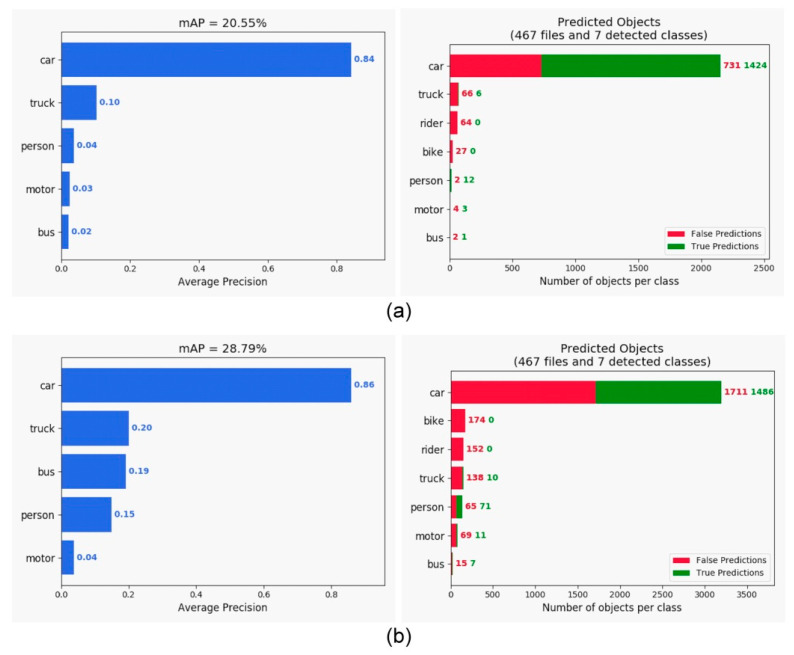
mAP comparison of the original RefineDet and our approach tested on our own dataset. (**a**) Evaluation of RefineDet. (**b**) Evaluation of our approach.

The comparison of the various detection algorithms tested on the PASCAL VOC dataset is shown in Table 1. Our approach provided the best mAP compared with that of RefineDet [17], SSD [10], YOLOv2 [11], Faster R-CNN [7], and R-FCN [8]. Moreover, it maintained a rate of 25 frames per second (FPS). Hence, we detected objects in real time. For the KITTI dataset, we simplified the number of classes from 16 to 3 (car, person, and bicycle) for the evaluation and sped up the frame rate to 50 FPS on the NVIDIA GTX 1080 platform, as shown in Figure 6. Comparative test results for RefineDet are presented in Figure 7, where the mAP of the proposed approach was better than that of RefineDet. In addition, our approach detected the person class more effectively. For the BDD100K dataset, seven classes were used for evaluation. This dataset contained more challenging scenes, such as those in low illumination or containing occlusions. The object detection evaluation is illustrated in Figure 8. The results showed that the mAP of the proposed approach was larger than that of RefineDet. Moreover, our approach performed better than RefineDet for detecting objects. Although the accuracy was lower compared with the KITTI dataset results, our approach exhibited the desired improvements. Finally, we collected our own dataset from Taiwan road scenes for evaluation. The image sequences were captured from a car recorder at 30 FPS with a resolution of 1280 × 720. The object detection evaluation in our dataset is illustrated in Figure 9. From the results, the mAP of the proposed approach was much better than that of RefineDet. In addition, our approach outperformed RefineDet for detection in each class.

### 4.3. Evaluation of Depth Estimation

The depth estimation network was trained with unsupervised learning and had a lower computational cost, which for the proposed fully convolutional neural network depended on the size of the input images. Using low-resolution images as inputs, we reduced the cost with rough depth estimation. Using PyD-Net [32] as a reference for the depth estimation network, we designed a light fully convolutional neural network with only six FPN layers for the image reconstruction loss, which reduced the complexity of Monodepth as well as the computational cost.

**Figure 10:**
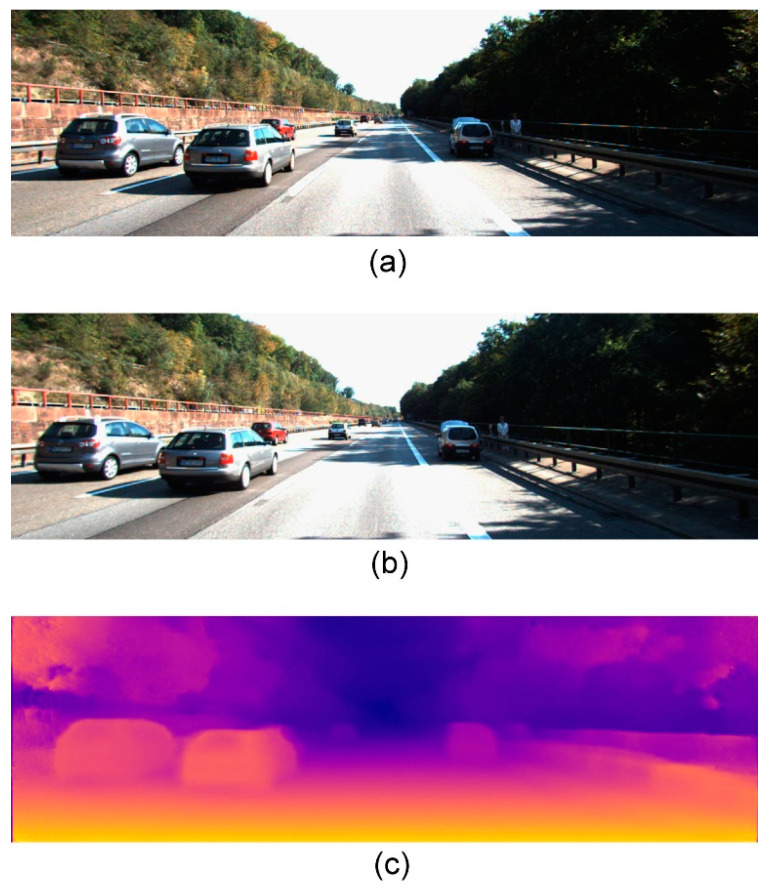
A stereo image pair and the estimated disparity map. (**a**) left image; (**b**) right image; (**c**) estimated disparity map.

**Figure 11:**
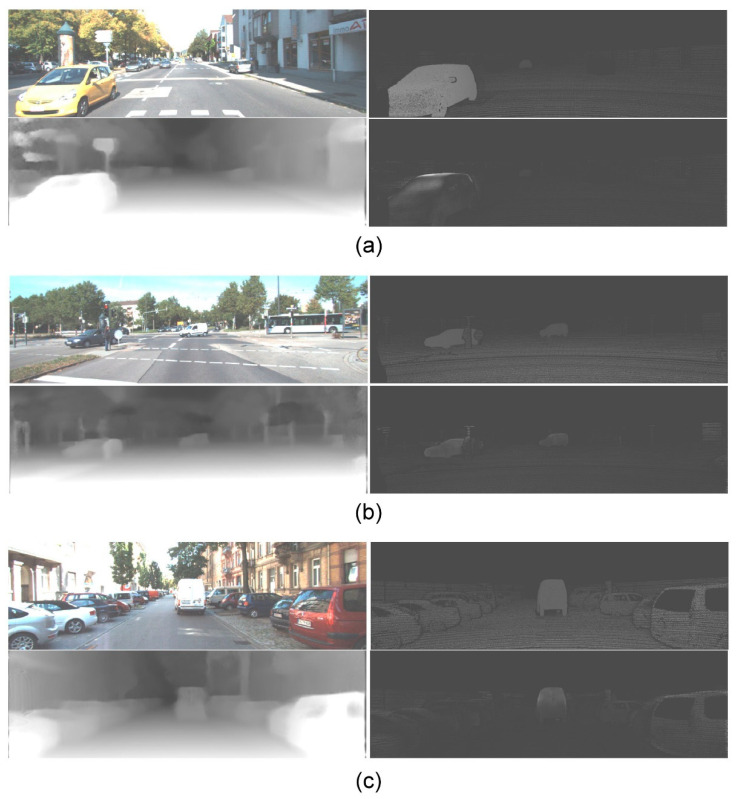
Three results of the depth map prediction using the KITTI dataset. (**a**) Traffic scene 1 with an approaching vehicle. (**b**) Traffic scene 2 with faraway small vehicles. (**c**) Traffic scene 3 with a vehicle for roadside parking.

The evaluation of the depth estimation network was performed with the KITTI dataset. A stereo image pair and the estimated disparity map are shown in Figure 10, which shows that the proposed approach can accurately estimate a dense depth map. Figure 11 shows several results of the depth map prediction with the input image (upper left), ground-truth disparity map (upper right), estimated disparity map (bottom left), and disparity difference between the ground-truth and prediction (bottom right). For traffic scene 1, there was an approaching vehicle and some objects. The depths of the vehicle and the objects were greatly estimated. For traffic scene 2, there were faraway small vehicles and some objects in the scene. The proposed approach estimated the depths well for the faraway small vehicles and the objects. For traffic scene 3, there was a vehicle for roadside parking and some objects in the scene, and the depths of the vehicle and the objects were reasonably estimated. These results showed that the proposed approach produces visually reasonable depth maps.

Moreover, we adopted common evaluation metrics. Let *P* be the number of pixels. The notations $d_{i}$ and $\overset{\wedge }{d_{i}}$ are the ground-truth disparity value and estimated disparity value, respectively. Each metric was defined in the followings. The root mean square error *RMS* was defined by

(11) $$RMS=\sqrt{\frac{1}{P}\sum_{i=1}^{P}{\left({\hat{d}}_{i}-d_{i}\right)}^{2}}.\;$$

The absolute relative difference *Abs-rel* was defined by

(12) $$Abs-rel=\frac{1}{P}\sum_{i=1}^{P}\frac{\left||{\hat{d}}_{i}-d_{i}|\right|}{d_{i}}.\;$$

The square relative difference *Sq-rel* was defined by

(13) $$Sq-rel=\frac{1}{P}\sum_{i=1}^{P}\frac{∥{\hat{d}}_{i}-d_{i}{∥}^{2}}{d_{i}}.\;$$

The root mean square logarithmic error *Log-rms* was defined by

(14) $$Log-rms=\sqrt{\frac{1}{P}\sum_{i=1}^{P}{\left(Log{\hat{d}}_{i}-Logd_{i}\right)}^{2}}.\;$$

The depth error ratio of one pixel *Er* was defined by

(15) $$Er=\;\max\left(\frac{d_{i}}{{\hat{d}}_{i}},\frac{{\hat{d}}_{i}}{d_{i}}\right)<t\;,\;\mathrm{where}\;t\;\epsilon \;\left[1.25,\;1.25^{2},\;1.25^{3}\right].\;$$

Finally, the evaluation metric *D1-all* was defined as the percentage of misclassified pixels (error > 3 pixels) in the whole image.

The comparison of different algorithms is shown in Table 2, where the proposed approach outperformed all previous methods on *Log-rms* and *D1-all* metrics. Our approach had similar accuracy as the methods of Lai et al. (stereo only) [35] and Godard et al. [12] + Stereo and was better than the other methods on *Abs-rel*, *Sq-rel*, and *RMS* metrics; however, it was less accurate on *Er* < 1.25 and *Er* < 1.25^3^ metrics. To summarize, the results indicated that the proposed approach had the same levels of error as the previous methods, and compared with the previous networks demonstrated its feasibility.

**Figure 12:**
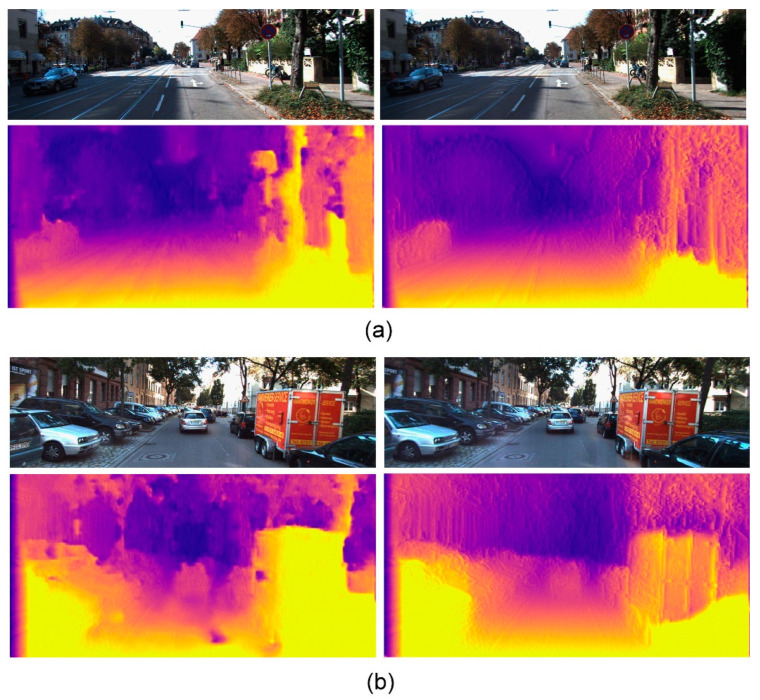
Disparity maps of two stereo image pairs generated using our approach (bottom left) display clear improvements over the original lightweight network (bottom right). (**a**) Scene 1. (**b**) Scene 2.

To accelerate the image processing, the resolution of each image was reduced to 512 × 256 pixels. Figure 12 shows the real-time processing results of two stereo image pairs. For each scene, the upper two images were stereo images. The disparity maps derived using our approach (bottom left) showed clear improvements compared with the disparity maps obtained from the original lightweight network (bottom right).

## 5. Conclusions

We presented an object detection and depth estimation approach based on deep learning techniques. Object detection was improved through the incorporation of the TCBs with the CNN as small objects were detected in real-time. Moreover, we applied binocular vision to the monocular-based disparity estimation network. The comparison with previous networks demonstrated the feasibility of the proposed approach. In future studies, object detection and depth estimation networks can be integrated into the feature extraction process within a convolutional network to reduce network and computational resources. Additionally, transfer learning will be used to enable the networks to train in object detection and depth estimation independently.

## Figures and Tables

**Table 1:** Comparison of detection algorithms tested on the PASCAL VOC dataset.

Approach	Zhang et al. [17]	Liu et al. [10]	Redmon et al. [11]	Ren et al. [7]	Dai et al. [8]	Our Approach
Backbone	VGG-16	VGG-16	VGG-16	ResNet-50	ResNet-50	VGG-16
Training Data	PASCAL VOC	PASCAL VOC	PASCAL VOC	PASCAL VOC	PASCAL VOC	PASCAL VOC
Input Size	320 × 320	300 × 300	416 × 416	320 × 320	320 × 320	320 × 320
Boxes	6375	6200	Unknown	Unknown	Unknown	6500
FPS	25	35	**67**	2.4	5.9	25
mAP	79.49	75.3	76.8	73.8	77.6	**79.75**

**Table 2:** Comparison of algorithms with various evaluation metrics.

Approach	*Abs-rel*	*Sq-rel*	*RMS*	*Log-rms*	*D1-all*	*Er <* 1.25	*Er <* 1.25^3^
Godard et al. [12]	0.124	1.40	6.137	0.217	30.350	0.841	0.975
Pilzer et al. [24] (half-cycle stereo)	0.228	4.277	7.646	0.318	Null	**0.748**	0.945
Pilzer et al. [24] (full-cycle+D+SE)	0.190	2.556	6.927	0.353	Null	0.751	0.951
Lai et al. (stereo only) [35]	**0.078**	**0.811**	**4.700**	Null	Null	0.983	Null
Poggi et al. [32]	0.153	1.363	6.030	0.252	Null	0.789	**0.630**
Godard et al. [12] + Stereo (no correlation)	0.083	0.944	4.765	0.163	13.087	0.927	0.986
The Proposed Approach	0.08	0.925	4.846	**0.160**	**12.480**	0.929	0.987

## Data Availability

Not applicable.
